# Issues of Data Acquisition and Interpretation of Paraseismic Measuring Signals Triggered by the Detonation of Explosive Charges

**DOI:** 10.3390/s21041290

**Published:** 2021-02-11

**Authors:** Józef Pyra, Maciej Kłaczyński

**Affiliations:** 1 Department of Mining Engineering & Occupational Safety, Faculty of Mining & Geoengineering, AGH University of Science and Technology, 30-059 Cracow, Poland; 2 Department of Mechanics and Vibroacoustics, Faculty of Mechanical Engineering and Robotics, AGH University of Science and Technology, 30-059 Cracow, Poland; maciej.klaczynski@agh.edu.pl

**Keywords:** vibration interference, acoustic wave, airblast shock wave, vibration transducers, acoustic pressure transducers, data acquisition

## Abstract

The paper tackles the issues of data acquisition during the measuring of vibrations caused by the detonation of explosive charges in various types of works (blasting in mines, demolition works, tunneling). Depending on the placement of an explosive charge (a charge detonated on the surface or a charge placed in a hole), it triggers side effects in the form of mechanical vibrations, which are propagated in the environment and may pose a hazard to buildings. In the case of propagation in the air, there is an acoustic wave and an airblast wave. For the assessment analysis on the impact of vibrations on buildings, a ground-propagated signal is used, while what is propagated by air is a disturbance. Selected examples in the paper demonstrate how an acoustic wave and an airblast wave interferes with the signal recorded by geophones. Afterwards, the paper presents the results of the tests conducted at a training area, during which various masses of explosive charges placed in different ways were detonated. The examples demonstrate that this interference may lead to the misinterpretation of recorded measurements. This paper is the first of two papers that will present the results of research into this matter and the suggested resolutions in order to eliminate this interference.

## 1. Introduction

The use of explosive charges in a wide range of sectors of the economy is inherently associated with effects resulting from their detonation. The most common effects that may have a negative impact on the environment include flyrock, airblast waves (shock waves) [1], acoustic waves, and ground vibrations [2]. All these effects may also have a negative impact on buildings located near the works involving the use of explosive charges [3]. Due to the negative effects of the detonation of explosive charges, other technologies are used to mitigate such effects, including mechanical mining in the mining industry [4], mechanical demolition of objects in the construction industry [5,6,7], or liquid carbon dioxide phase change fracturing technology (LCPCFT) [8,9].

Effects such as vibrations, airblast waves, and acoustic waves may overlap as a result of them being a physical phenomenon. Waves, which propagate in different media (ground, water, air) at different speeds, occur especially when explosive charges are detonated on the surface or under a small cover, i.e., during engineering and demolition works (e.g., explosive metal cladding, demolition works using shaped charges, etc.). It also can occur when blasting works in open-pit mines have been performed incorrectly (too small distance from an explosive charge in a blasthole to the nearest free or open face -burden, too short inter material used in the collar part of the blasthole to confine the gases from the detonation-stemming), or the location of the detonating explosive charges has not been examined sufficiently (caverns, zones with the lower burden—the crater effect) [10,11]. The interaction of these effects may lead to a change in the intensity of one effect or to a limitation in accuracy of the measurements while mitigating another effect. Thus, effects that interact with each other may also be called “combined effects” [12,13]. This issue is very important, notably when the measurements of an effect are made in close proximity to the location of the detonated explosive charge.

The authors of the paper [14] state that in the case of the detonation of explosive charges on the ground surface at short distances, the main effect is triggered by an airblast wave; at longer distances, the effect propagated by the ground becomes predominant, while at very long distances these dual effects may be analysed separately. The papers [15,16] tackle the issues of the effects of vibrations and airblast waves triggered by the detonation of explosive charges in respect to the protection of the environment and people. However, they lack information on the possibility of a simultaneous occurrence of an acoustic wave propagated in the air or the possibility of its interference in the seismic signal.

The paper [17] analysed the correlation of ground vibrations and the pressure of an airblast wave recorded in close proximity of the building, to an acoustic wave being recorded in the rooms. The results indicated that the vibrations recorded subsurface in the immediate vicinity of the building triggered an acoustic wave inside the building, while the pressure of the airblast wave had no impact on the newly created acoustic wave. Furthermore, the paper [18] used examples of high-energy impulse noise measurements to demonstrate the issue of its efficacy in its distribution at a greater distance from blasting works performed in an open-pit mine, as well as at a training area. Emphasis was placed on the uncertainty of the measurements in terms of the evaluation of the effect on the acoustic climate. In particular, the impact of meteorological conditions (wind strength and direction, clouds, temperature, humidity, and atmospheric pressure) on the measurements was discussed.

Advanced modelling tools based on neural networks are used to study the propagation of vibrations on the ground or of an airblast wave in the air [19,20,21,22,23,24]; Albeit, they do not factor in the possibility of interference by one medium to another with the results of their measurements.

The authors of the paper [25] noticed that the source of interference of a seismic signal is environmental noise, e.g., the detonation of explosive charges, wind sound, sounds of traffic, or even ocean waves. The problem occurs when the frequency structure of a seismic signal is similar to the frequency structure of the interference or when the seismic signal is weaker than the interference. Accounting for this, the authors of the papers [25,26,27] analysed the possibility of air noise reduction for seismic records. To do so, they used microphones so that their records can be subsequently utilized as a filter to separate the seismic signal.

The issues associated with the effect of airblast waves and ground vibrations and their mutual correlations are relevant today, especially in terms of assessing their effect on nearby buildings where occupancy may exist. The issue is in the separation of an airblast wave and an acoustic wave during the measuring process, which should be analysed differently using appropriate measurement and analytical procedures [28,29,30]. Therefore, the further part of the paper includes examples of vibration (ground and building) and airblast wave recordings made during the various occurrences of using explosive materials, followed by the results of field tests (at a training area).

## 2. Issues in the Acquisition of Data with Signals of Vibrations Triggered by the Detonation of Explosive Charges

The basis for starting the analysis of recorded signals is to make proper measurements of a given phenomenon (data acquisition). For effects triggered by the detonation of explosive charges, this is of particular importance because such records are used to determine safe masses of explosive charges for mines or to assess the impact on buildings near the location of detonating such charges. When measurements are incorrect, results may be misinterpreted and thus, lead to a possible inaccurate determination of safe masses of explosive charges or to an incorrect assessment of the impact of vibrations on a building causing unnecessary destruction to property.

Given that this study was performed in Poland, vibrations were measured according to PN-B-02170:2016-12 [31]. However, due to the lack of guidelines in Poland for measuring airblast waves, guidelines were made according to the presented papers of [32,33].

In each case, measurements were made by UVS 1608 manufactured by Swedish company Nitro Consult AB or by VIBRALOC manufactured by Swedish company ABEM Instrument AB, featuring three-component geophones and broadband microphones and in some cases, meters, sound, and vibration analysers SVAN 958 by SVANTEK.

### 2.1. Equipment Used to Measure Vibrations and Airblast Wave

The UVS 1608 vibration meter is an eight-channel system used to record ground vibrations (Table 1). It features an 11-bit AC/DC converter, which allows signals to be recorded at approx. 66 dB. The built-in software makes it possible to filter waveforms using low and high-pass filters and to determine vibration parameters for individual directional components in addition to the actual speed vector.

The VIBRALOC recorder is a multichannel device, featuring digital recording and is used to monitor ground vibrations, airblast wave, or vibrations in buildings.

The microphones used in the study (UVS Airblast Microphone 4312 by ABEM Instrument AB, formerly Nitro Consult AB) are electret (polarised) microphones and their parameters are shown in Table 2.

**Figure 1:**
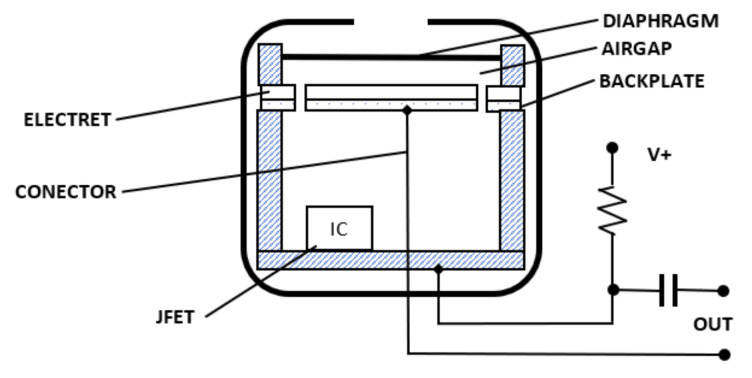
Cutaway view of a typical electret condenser microphone.

The mechanical-electrical diagram of a condenser microphone with internal polarization is shown in Figure 1. In such microphone, the diaphragm is a polyester film, teflon, or a similar material that is metallised, usually by gold spraying, to form a conductive surface, which is grounded by a metal washer. Even though the diaphragm may be a charged electret surface, the design is often referred to as a “back electret”. This is due to an electret film, which maintains a high voltage (often 50 volts or more) for many years and is applied to the back-metal plate. The movement of the diaphragm in relation to the polarised back plate causes changes in voltage ±∆U, producing electromotive force. Low microphone sensitivity (approx. 1–5 mV/Pa) means that it is sensitive to an airblast wave in the low to medium frequency range (from approx. 1 Hz to approx. 10 kHz). The capacitor of a condenser microphone, which is called a transmitter, is a high internal impedance voltage source of a capacitive nature, to which a cable cannot be connected directly. That is why the capsule contains an integrated preamplifier, which converts high capsule impedance (several GΩ) to low microphone output impedance (>1 kΩ). Importantly, in this type of microphone, electromotive force is proportional to the inclination of the diaphragm and not to speed. Consequently, for a condenser microphone to have constant electromotive force at the same pressures but a different wave frequency, the diaphragm inclination as a function of frequency must be constant. This means that the diaphragm vibration velocity must be directly proportional to the frequency.

Vibration velocity transducers used in the study (UVS Geophone DIN 4101 vertical, 4102 horizontal, 4103 triaxial) are electrodynamic transducers with resonant frequency compensation and provide a linear response characteristic from 1 Hz (Table 3).

In general, three types of transducers are used to measure vibrations, i.e., seismometers—for vibration displacement amplitude measurements; geophones—for vibration velocity amplitude measurements; and accelerometers—for vibration acceleration amplitude measurements. It is assumed that the seismometer is a tool for seismological purposes and is used to observe shocks, whereas the geophone is a tool for seismoacoustic purposes and is used to observe vibroacoustic events.

**Figure 2:**
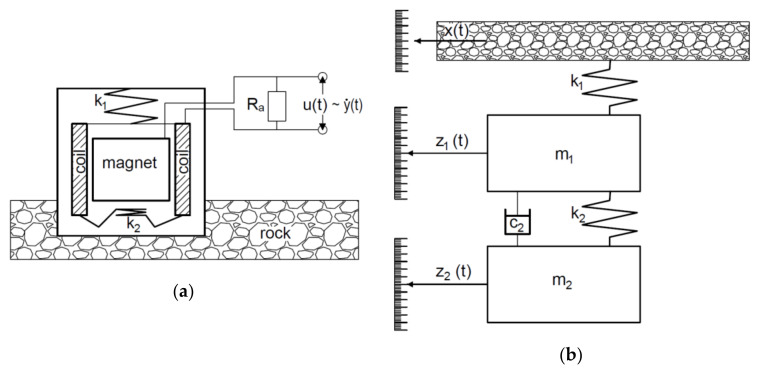
Diagram and principle of operation of the electrodynamic geophone: (**a**) simplified “electrical” diagram, (**b**) mechanical equivalent diagram.

**Figure 3:**
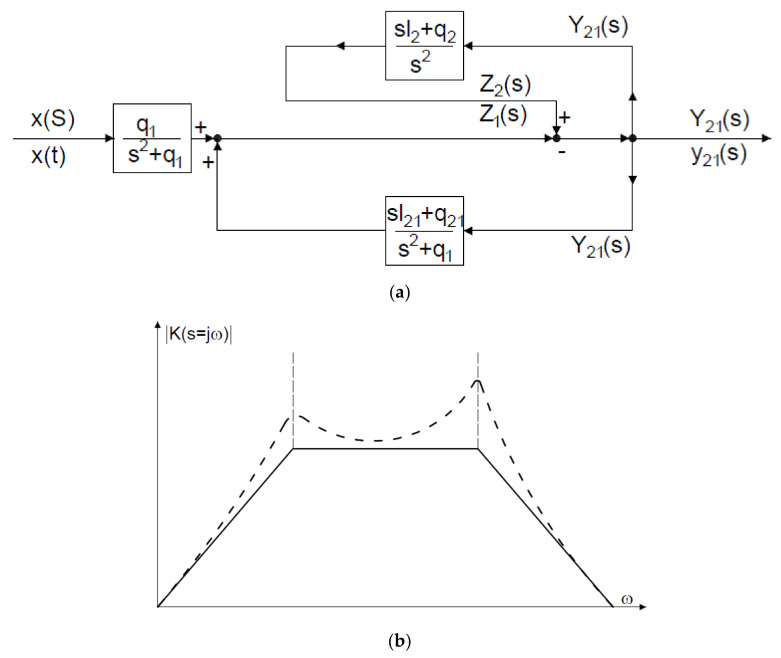
(**a**) Block diagram of the geophone, variables x, y, z correspond to displacements for three directions (**b**) amplitude characteristic for different attenuation values.

Contrary to the most common information in literature, the geophone is designed as shown in Figure 2a and includes, in addition to the housing, two springs, a moving coil, and a moving magnet, which means that it has two degrees of freedom. The voltage output signal *u(t)* is proportional to the velocity of vibrations of the coil in relation to the magnet, and-when the electrical diagram is replaced with a mechanical model-the energy-absorbing external load (*R_a_*) should be replaced with a Newtonian damper (*c*_2_). Figure 2b is a mechanical analogy of the electrical diagram shown in Figure 2a, whereas Figure 3a presents a block diagram of signal flow and Figure 3b shows a typical amplitude-frequency characteristic. Figure 2b shows that the geophone only responds to the axial component of forced vibration, thus *x(t)* is a displacement of the vibrating elements of the ground on which the sensor is located. *z*_1_*(t)* and *z*_2_*(t)* are displacements of the masses *m*_1_ and *m*_2_ in relation to their equilibrium. Electromotive force *U(t*) at the geophone output is relatively proportional to the speed of the winding in relation to the magnetic field. A system of equations for the geophone mechanical model is expressed as (1):

(1) $$\left\{\begin{array}{c} {\ddot{z}}_{2}\left(t\right)=\frac{c_{2}}{m_{2}}{\dot{y}}_{21}\left(t\right)+\frac{k_{2}}{m_{2}}y_{21}\left(t\right)\\ {\ddot{z}}_{1}\left(t\right)=\frac{c_{2}}{m_{1}}{\dot{y}}_{21}\left(t\right)+\frac{k_{1}}{m_{1}}\left(x\left(t\right)-z_{1}\left(t\right)\right)+\frac{k_{2}}{m_{1}}y_{21}\left(t\right) \end{array}\right.$$

where:

$y_{21}\left(t\right)=z_{2}\left(t\right)-z_{1}\left(t\right)$—relative displacement,

${\dot{y}}_{21}\left(t\right)={\dot{z}}_{2}\left(t\right)-{\dot{z}}_{1}\left(t\right)$—relative velocity,

*c*_2_—attenuation of magnet suspension in relation to the coil,

*k*_1_—elasticity of the suspension of the measuring system in the housing,

*k*_2_—elasticity of magnet suspension in relation to the coil.

Taking the following symbols and introducing a transfer function, the system of Equations (1) takes the following form:

(2) $$\left\{\begin{array}{c} Z_{2}\left(s\right)=s^{-2}\left(sl_{2}+q_{2}\right)Y\left(s\right)\\ Z_{1}\left(s\right)=\frac{q_{1}}{s^{2}+q_{1}}X\left(s\right)+\frac{sl_{21+q_{21}}}{s^{2}+q_{1}}Y\left(s\right) \end{array}\right.$$

Using (2) and assuming that the relative displacement of sensor components in a transfer function takes the form *Y*(*s*) = *Z*_2_(*s*) − *Z*_1_(*s*), (3) is obtained.

(3) $$Y\left(s\right)=\frac{-s^{2}q_{1}}{s^{4}-s^{3}\left(l_{2}-l_{21}\right)+s^{2}\left(q_{1}+q_{21}-q_{2}\right)-sl_{2}q_{1}-q_{1}q_{2}}X\left(s\right)$$

The function expressed as the Equation (3) expanded into partial functions is given in Figure 3a.

Equation (3), being a transfer function of the geophone-type transducer, combines the response *Y*(*s*)~*x*(*t*) with the force *X*(*s*)~*x*(*t*). If the attenuation of magnet suspension in relation to the coil *c*_2_ is small or the load *R_a_* on this sensor is very high, *l*_2_ and *l*_21_ move towards 0, while the equation is significantly simplified, taking the form of (4).

(4) $$Y\left(s\right)=\frac{-s^{2}q_{1}}{s^{4}+s^{2}\left(q_{1}+q_{21}-q_{2}\right)-q_{1}q_{2}}X\left(s\right)$$

**Figure 4:**
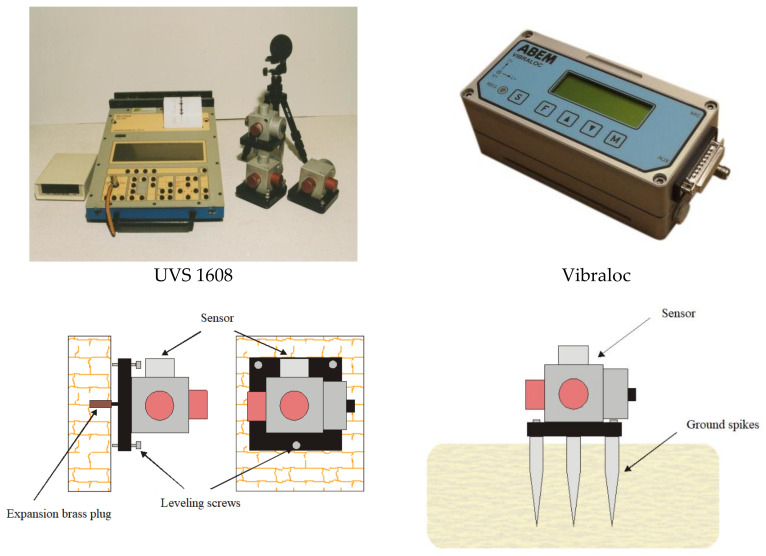
Vibration and airblast wave meters and the method of fixing the sensors to the foundation in the ground.

For geophones, the attenuation of suspension *c*_2_ is selected sufficiently small and the parameters of *k*_1_, *k*_2_, *m*_1_, and *m*_2_ are selected so that there are two real and negative solutions to a biquadratic equation of the function in the function denominator (4). The result is that the Nyquist plot has four poles, while poles with positive pulsation has two resonant frequencies for the amplitude characteristic shown in Figure 3b. It is the existence of two poles that determines the appearance of the seismometer’s characteristic (one resonant frequency). Laboratory measurements of the amplitude characteristic confirm the existence of two poles although their sharpness, especially of the low-frequency pole, may depend on the type (or even model) of geophone. The frequency band in which the characteristic is approximately flat, Figure 3b, is approx. 30–800 Hz, while the use of compensation makes it possible to increase the range to 1–1000 Hz. The issue of the geophone response to short-term force when attenuation is different from zero is still an area of research. Figure 4 shows the equipment used for measurements: UVS 1608—an eight-channel device and Vibraloc—a four-channel device (frequency range 2–250 Hz) and the method of fixing the sensors to the foundation in the ground.

The next part of the paper includes examples of various records of vibration and airblast wave signals-including their Fast Fourier Transforms FFTs are calculated over the entire signal in Matlab)—during the performance of works with explosive materials.

### 2.2. Results of Vibration and Airblast Wave Pressure Measurements for Different Works Using Explosive Materials

**Figure 5:**
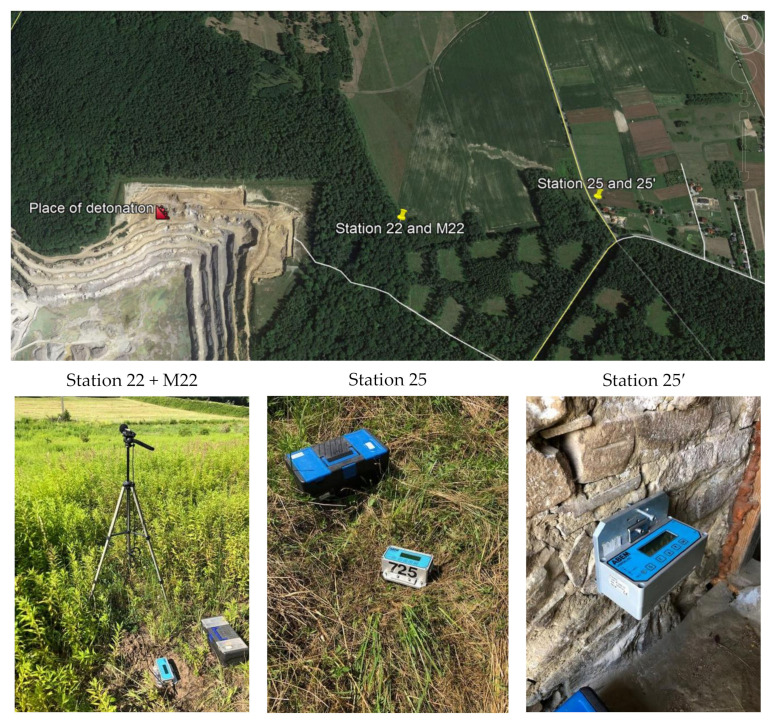
Blasting site and measurement stations.

**Figure 6:**
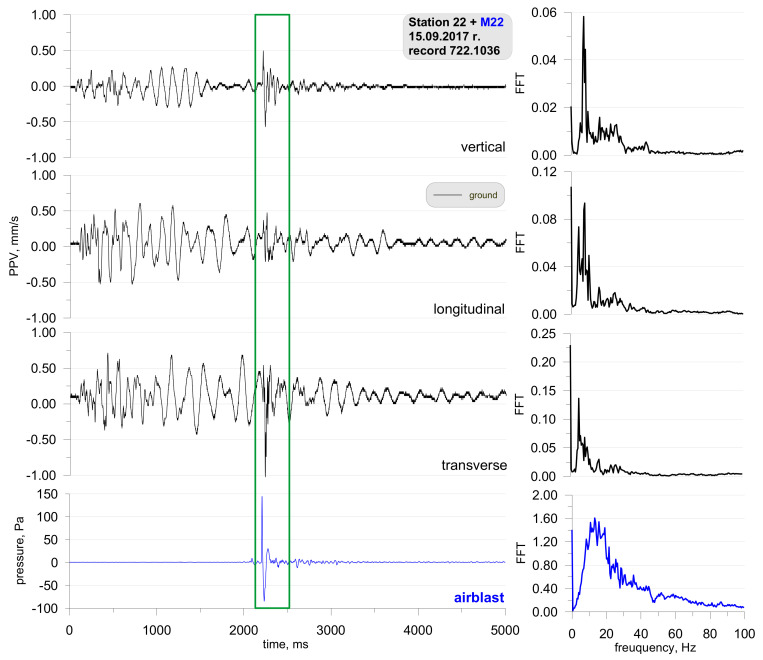
Ground vibration seismogram (three directional components) and recordings of airblast wave pressure combined with the analysis of the FFT-Station 22.

**Figure 7:**
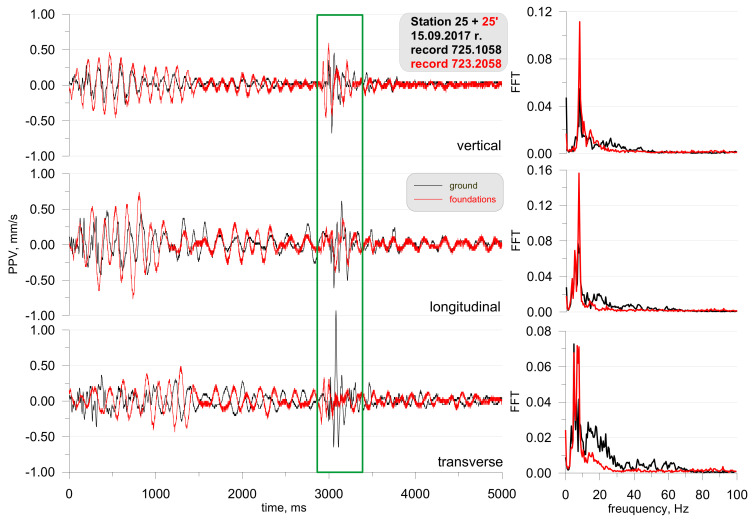
Seismogram of the vibration of the ground and building foundation (three directional components) combined with the analysis of the FFT-Station 25.

Figure 5 shows the location of the measuring stations, and Figure 6 and Figure 7 show a record of the vibration of the ground, building foundation and airblast wave triggered by blasting works in an open-pit mine. Station 22 (measuring the ground vibration and airblast wave-sensor anchored in the ground in accordance with the manufacturer’s instructions and microphone placed 1.5 m above the ground directly above the sensor) and Station 25 (measuring the vibration of the ground and building foundation) were located 630 m and 1150 m away from the blasting site, respectively. The first sensor anchored in the ground in accordance with the manufacturer’s instructions and second sensor rigidly attached to the foundation at ground level. The building was a typical single-family building with a brick structure. Station 25 was located in the same research profile (identical propagation direction) as Station 22 (geological cross-section of the sites is not known). There was a natural obstruction in the form of a forest on the airblast wave propagation path (Figure 5). Bench blast with a high bench was used, with a total of 1349 kg of explosive materials being detonated. The maximum mass of an explosive charge per millisecond delay time was 149.9 kg.

The analysis of both figures (Figure 6 and Figure 7) reveals that the airblast wave pressure interfered with the geophones. Unfortunately, due to a small number of microphones, Station 25 did not have a microphone and yet, based on the record from Station 22 it can be concluded that the interference in the second part of the signal (t = 3050 ms) was caused by an airblast wave. More importantly to note was that of the airblast wave; the effect of the airblast wave had triggered higher vibrations than those propagated in the ground. In the absence of airblast wave measurements during practical operations, the cause of such interference would not have been identified, which would result in an incorrect determination of propagation equations and an incorrect evaluation of the effects on the building. The frequency structure is characterised by lower frequencies in the range up to 20 Hz. It should also be emphasised that the airblast wave pressure of approx. 150 Pa at a distance of over 630 m from the blasting site in an open-pit mine is very rare. In such situations, the airblast wave pressure is not expected to exceed 20 Pa.

**Figure 8:**
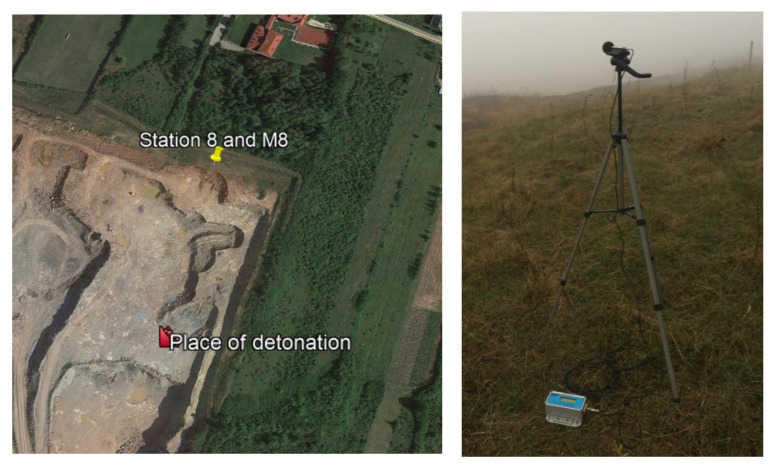
Blasting site and measurement stations.

**Figure 9:**
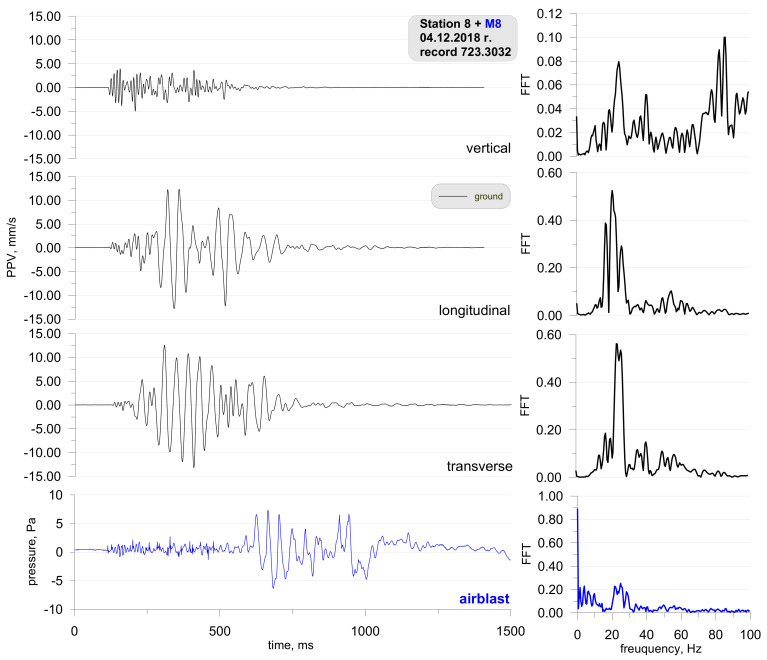
Ground vibration seismogram (three directional components) and recordings of the airblast wave pressure combined with the analysis of the FFT-Station 8.

It is not always the case that blasting works generate such a high airblast wave pressure. For illustrative purposes, Figure 8 shows the location of the measuring stations, and Figure 9 shows the record of the vibration of the ground and airblast wave made during blasting works in another open-pit mine. In that case, the measurement station (sensor anchored in the ground in accordance with the manufacturer’s instructions and microphone placed 1.5 m above the ground directly above the sensor) was much closer—approx. 167 m from the detonation site and close to the excavation edge (no obstructions were along the airblast wave propagation path—Figure 8). Bench blast with a high bench was used, during which a total of 1197 kg of explosive materials were detonated and the maximum mass of an explosive charge per delay period was 28.5 kg.

Despite a small distance from the detonation site, the pressure value is significantly lower than in the first case (Figure 6). It can also be seen that the airblast wave was recorded much earlier (t = 650 ms) and there is no clear pressure jump. What is more, the record of the airblast wave displays small values right from the start. This was probably caused by an acoustic wave triggered from the detonation of the connector surface (fuses in a plastic body). Frequencies for horizontal components (longitudinal and transverse components) are in the range of 20–40 Hz. Frequencies for the vertical component on the other hand are higher—70–90 Hz. 

Despite the much shorter distance between the place where the blasting works are performed and the measuring point (example from Figure 9), the use of explosives did not disturb the seismic signal recorded by the geophone, as was the case at the measurement points presented in the example in Figure 6 and Figure 7. Probable cause excitation of high pressure of airblast was badly performed blasting works (too small burden, too short stemming) or a cavern in which the concentration of the explosive charge could take place (crater’s effect).

**Figure 10:**
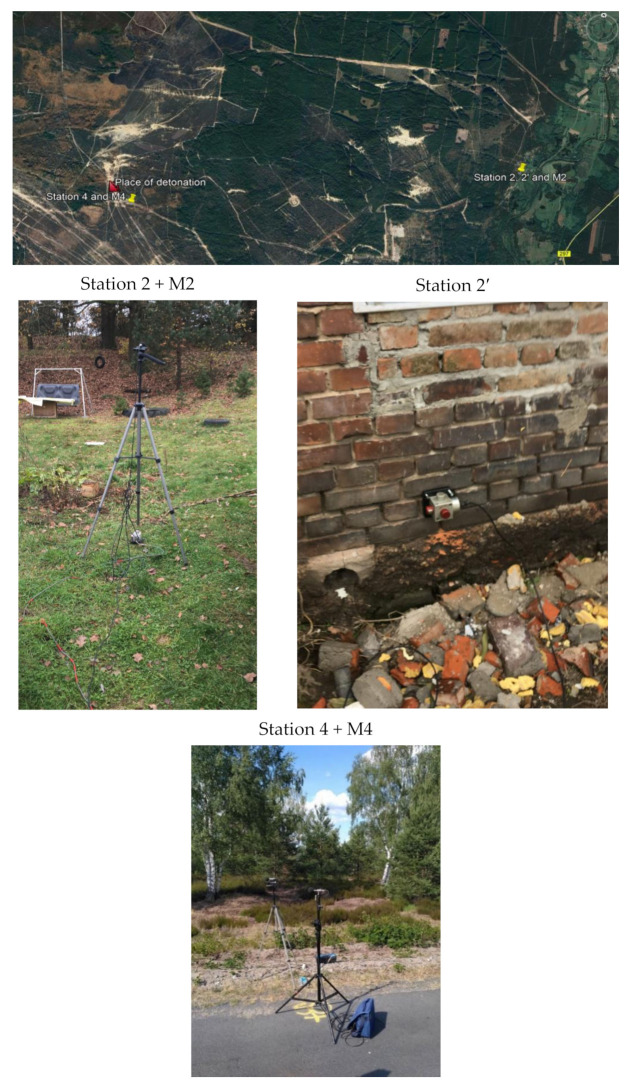
Blasting site and measurement stations.

**Figure 11:**
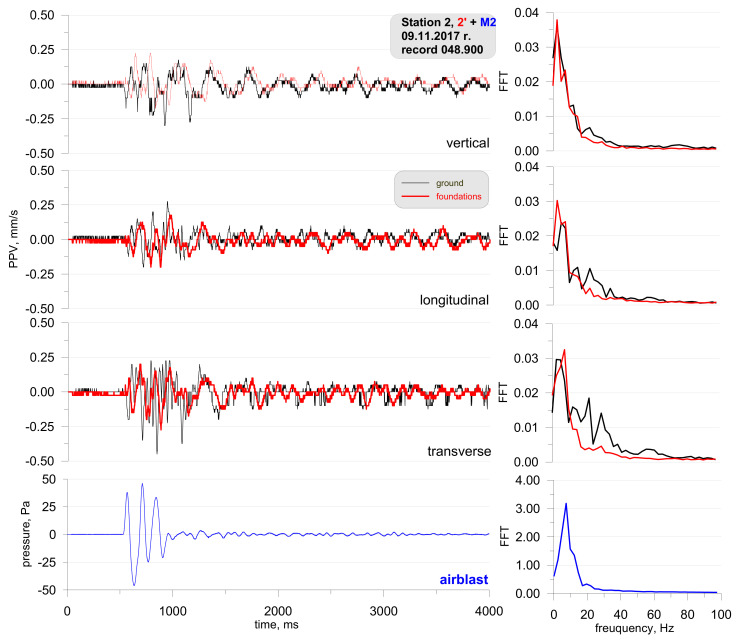
Seismogram of the vibration of the ground (three directional components), foundation, and record of airblast wave pressure together with the analysis of the FFT-Station 2.

**Figure 12:**
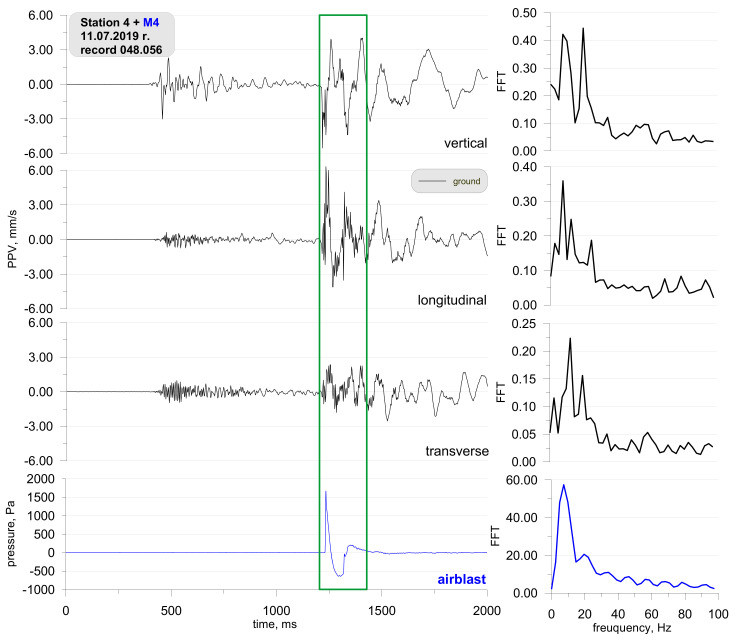
Ground vibration seismogram (three directional components) and recordings of airblast wave pressure together with the analysis of the FFT-Station 4.

Figure 10 shows the location of the measuring stations, and Figure 11 and Figure 12 present the vibration measurement results recorded on the ground, foundation, and airblast wave during metal cladding with the use of explosive materials. The first sensor anchored in the ground in accordance with the manufacturer’s instructions and second sensor rigidly attached to the foundation at ground level. A microphone placed 1.5 m above the ground directly above the sensor. The building was a typical single-family building with a brick structure. For this analysis, measurements were taken at a distance of 6500 m (Figure 10) from the location where 450 kg of explosives were simultaneously detonated on the surface. The measurement results are shown in Figure 11. On the other hand, Figure 12 shows the measurement results taken from the station located at a distance of 295 m (Figure 10) when 400 kg of explosives were simultaneously detonated. In both cases, the main propagation medium was the air.

Figure 11 shows the results of the measurements taken at one of the stations during the first study phase. The main objective was to verify the effect of the detonation of explosive charges on buildings near the training area. The records show that vibrations and the airblast wave were recorded at the same time (by one 8-channel measuring device) so there were no ground-propagated vibrations (the entire explosive charge was detonated on the surface so it was impossible for it to trigger vibrations that would propagate at a distance of 6500 m). The recordings therefore were caused by pressure. A question then arises, did the airblast affect the geophones ability to record the findings properly or is it the effect of the airblast wave pressure on the ground and the building?

The second phase of the study focused on the determination of safe areas for people who performed metal cladding works. The detonation of explosive charges caused ground vibrations (recorded in the range of 500–1250 ms—Figure 12), which was recorded at a 295-metre distance from the detonation site; however, their value is not comparable to that of the effect caused by the airblast wave (recorded at over 1250 ms). The characteristic frequency for the airblast wave and ground vibrations was 10 Hz and 10–30 Hz respectively. In this case, the same question may be asked: what did the airblast wave pressure affect (Did the airblast affect the geophones ability to record the findings properly, or is it the effect of the airblast wave pressure on the ground)?

**Figure 13:**
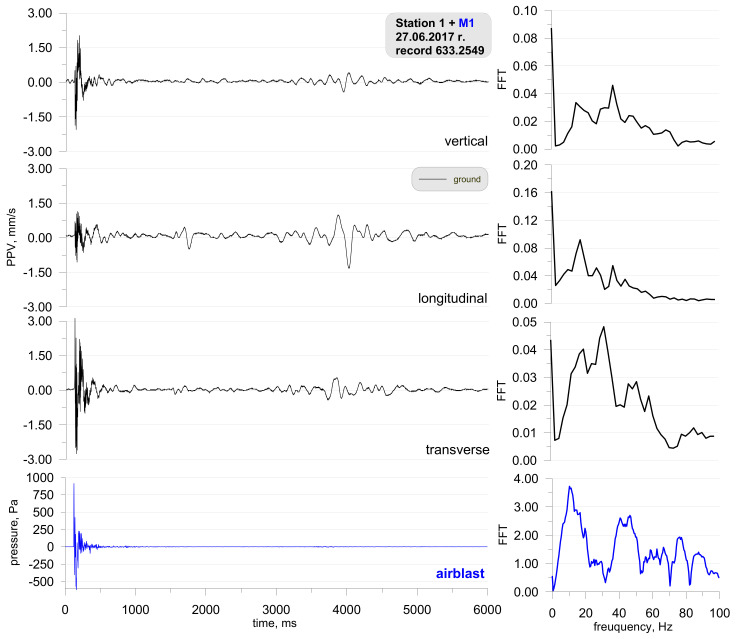
Ground vibration seismogram (three directional components) and record of airblast wave pressure, together with the analysis of the FFT-Station 1.

Another example involves recording the effect observed when cutting a steel structure with the use of shaped charges located at a height of approx. 10 m above ground level. The total mass of the charge that was simultaneously detonated was 4.6 kg, while the distance between the detonation site and the measurement station was approx. 80 m in a straight line. The results of the measurements at one of the stations are shown in Figure 13 (sensor anchored in the ground in accordance with the manufacturer’s instructions and microphone placed 1.5 m above the ground directly above the sensor, there was no air obstruction between the detonation site and the measuring station).

In spite of the low mass of the explosive material, the detonation of shaped charges generates a very high airblast wave pressure compared to Figure 11. The airblast wave pressure could have interfered with the geophones again because the ground vibrations start at the same time as the airblast wave recording. Likewise, it could also be that the pressure from the airblast created vibrations in the ground. Ground-propagated vibrations occur in the record shown in Figure 13 for the time above 3500 ms, i.e., when the steel structure falls to the ground. The frequency response for the airblast wave differs from the previous examples except for the frequency of 15 Hz; there are also higher frequencies—45 and 75 Hz.

**Figure 14:**
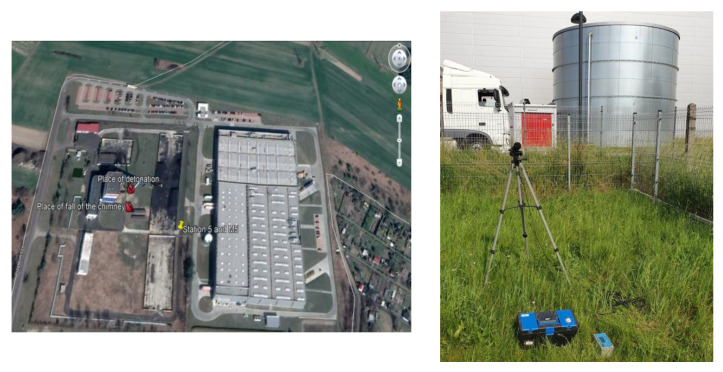
Blasting site and measurement stations.

The penultimate example covers the results of the measurements taken at one of the stations (Station 5) during the demolition of an 80-m reinforced concrete chimney using explosive materials (Figure 14). The distance from the measurement station to the detonation site was 98 m and to the location where the centre of gravity of the chimney fell was 80 m. A total of 11 kg of explosives were used to demolish the chimney. Sensor anchored in the ground in accordance with the manufacturer’s instructions and microphone placed 1.5 m above the ground directly above the sensor. There was no air obstruction between the detonation site and the measuring station.

**Figure 15:**
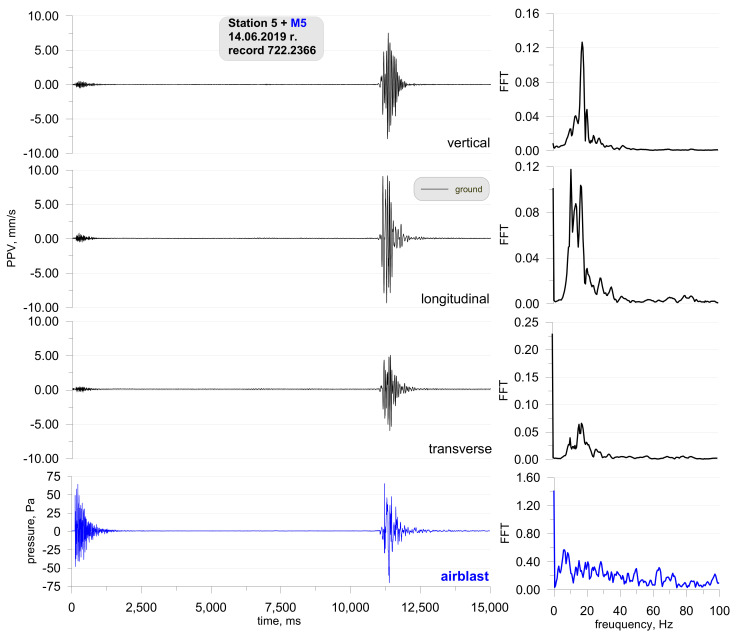
Ground vibration seismogram (three directional components) and recording of airblast wave pressure combined with the analysis of the FFT-Station 5.

Two phases can be clearly distinguished in the record shown in Figure 15. The first one is associated with the detonation of explosive charges (0–2000 ms) where ground vibrations are small, whereas the other (from 11,500 ms) is associated with the fall of the chimney to the ground. Ground vibrations are considerably greater in the latter phase. Both the detonation of explosive charges and the chimney fall generated an airblast wave having a pressure of approx. 70 Pa. Both phases may be analysed separately in this case.

**Figure 16:**
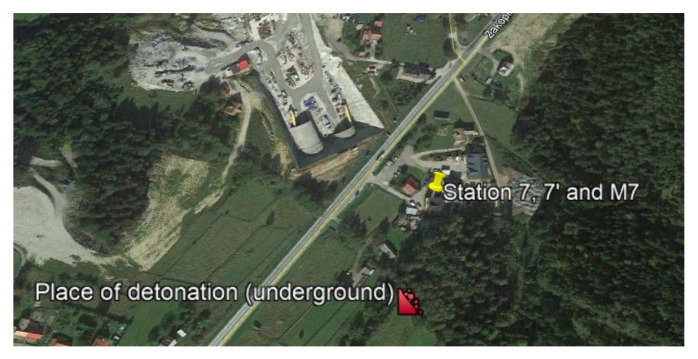
Blasting site and measurement stations.

The last example includes the recordings from one of the measurement stations (Station 7) during blasting works associated with tunnelling. In order to demonstrate that there was no airblast wave occurring above ground, the station was located on the surface above the blasting site and at a distance of approx. 160 m (Figure 16). The recorders were placed on the ground and in a building. The first sensor anchored in the ground in accordance with the manufacturer’s instructions in front of the building and second sensor rigidly attached to the foundation at ground level inside the building. The building was a typical single-family building with a brick structure. The microphone for airblast wave measuring was located in a closed building, 1.5 m above the sensor. The recordings show that the vibrations and pressure changes were caused by propagation in the rock medium only. For the blasting works, the mass of the explosive charge in a single hole was 3–5 kg at a 24–45 kg per millisecond delay period and the total mass for the face was 658 kg. The geological cross-section of the sites is not known.

**Figure 17:**
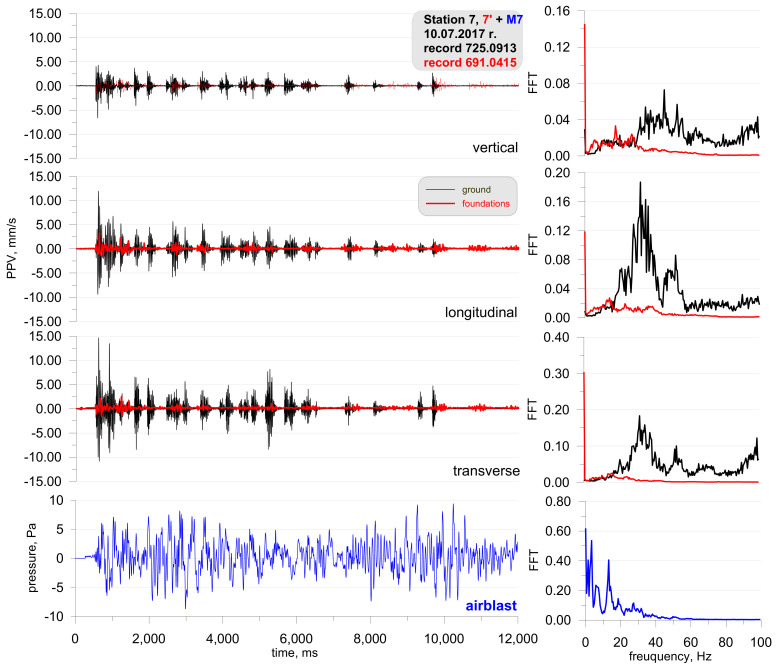
Seismogram of the vibration of the ground, foundation, and recording of airblast wave pressure combined with the analysis of the FFT-Station 7.

The analysis of Figure 17 revealed that the vibrations recorded in the ground were much greater than the vibrations recorded in the building foundation. Frequencies of over 30 Hz dominate the vibration structure for both the ground and the foundation; frequencies for the microphone recorded were 5 and 18 Hz. The microphone recording is interesting because, as already mentioned, the microphone was inside a closed building. In addition, the signal was only obtained from the ground-propagated vibrations (underground blasting works were at a depth of approx. 85 m). Therefore, it can be assumed that the microphone recorded an acoustic wave caused by vibrations.

## 3. Diagnostic Testing of Equipment under Field Laboratory Conditions

Due to the uncertainty of the influence of vibrations and interference to the geophones, tests in field conditions were performed at a training area for testing purposes as well as in a reverberation chamber. Equipment was included for measuring sound pressure levels in the 1 Hz–20 kHz band.

**Figure 18:**
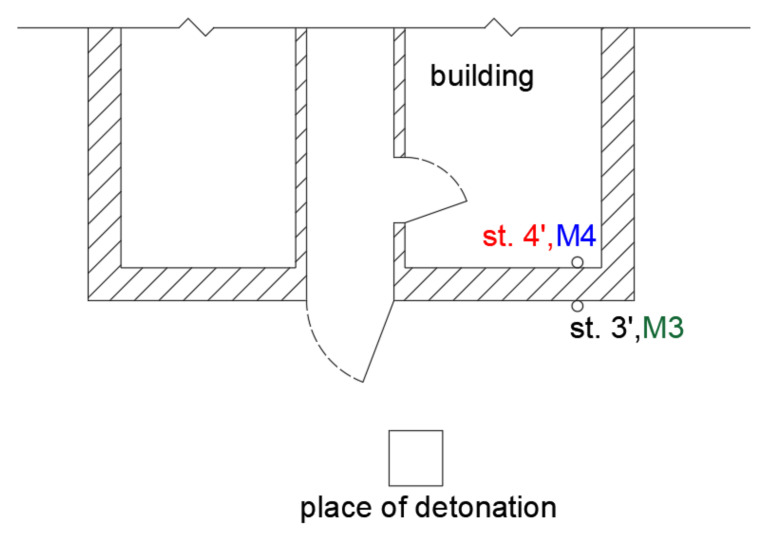
The sketch of location of measurement stations and explosives detonation.

**Figure 19:**
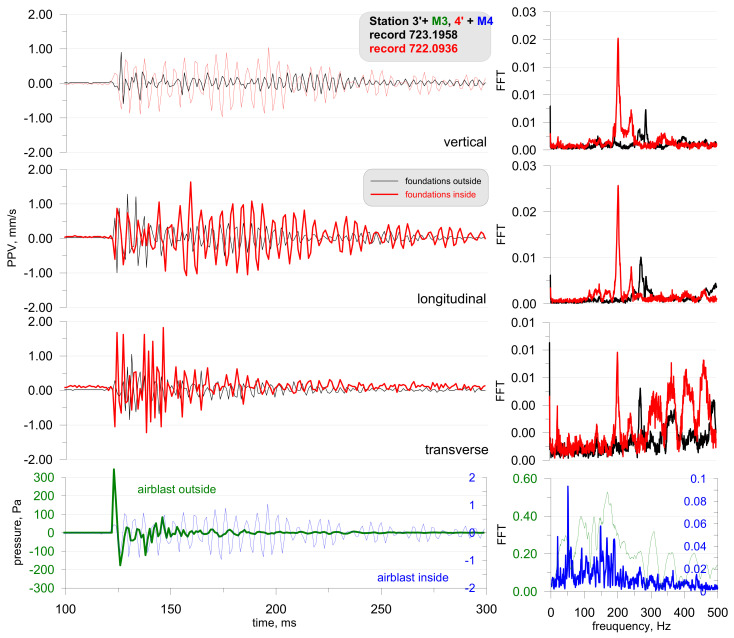
Foundation vibration seismogram (three directional components) and recordings of airblast wave pressure combined with the analysis of the FFT-Station 3 and 4.

The first phase involved measurements in field conditions, during which, small pyrotechnic charges were detonated followed by the detonation of larger explosive charges. These measurements were taken on the foundation of a building both inside (Station 3) and outside (Station 4)—on either side of the wall (Figure 18). The airblast wave pressure on both stations was also measured during the measurements of building vibrations. The effect was triggered by a small mass of pyrotechnic charge (approx. 3 g), which was suspended at a height of 0.6 m. The distance to the measuring stations was 5 m. All building windows and doors were closed during the measurement. The measurement results are shown in Figure 19.

The analysis of Figure 19 reveals that the sensor located outside the closed building (in black) recorded lower vibrations than the sensor located inside (in red). The findings are completely different for records made by the microphones. The microphone located outside (in green) recorded a very high pressure, exceeding 300 Pa, while the microphone inside recorded a pressure of only 1 Pa. The frequency structure of the signals from both the geophones and the microphones were dominated by high frequencies in the range of 200–300 Hz (tests were performed several times—with the same effect). Such high frequencies are untypical of buildings. What is more, the vibration velocity values are very high when one considers the mass of the pyrotechnic charge suspended at height. The recordings made by the geophones may point to the impact of an acoustic wave on the geophones. The wave phenomenon that arose inside the building was probably caused by the formation of an acoustic wave (reverberation), hence such a long recording signal from the internal microphone. The difference in the recordings of vibrations by geophones may indicate the probable influence of the acoustic wave on the geophones themselves. If it were the vibrations of the object, the records inside and outside should be practically the same.

**Figure 20:**
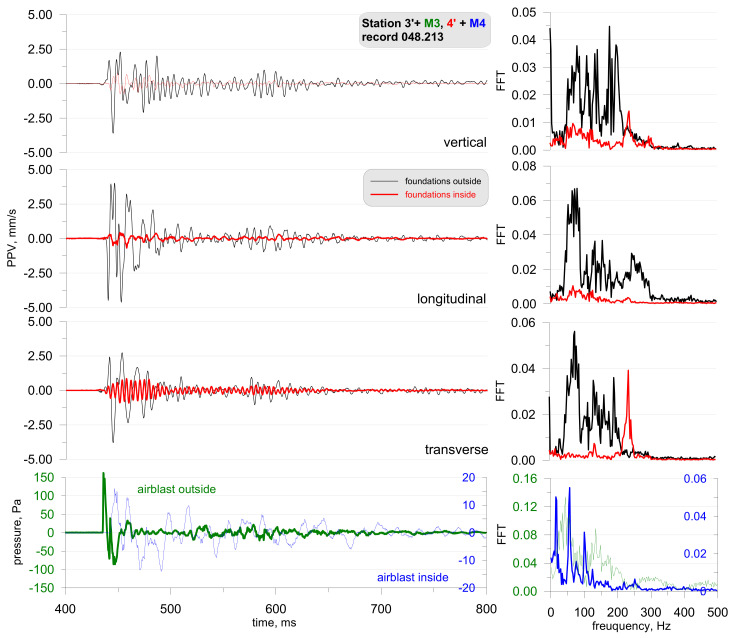
Foundation vibration seismogram (three directional components) and recording of airblast wave pressure combined with the analysis of the FFT-Station 3 and 4—explosive charge on the surface.

**Figure 21:**
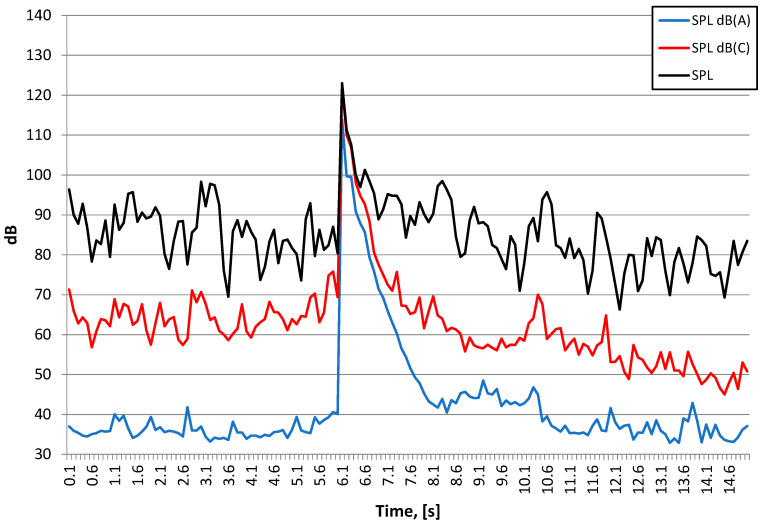
Waveforms of Sound Pressure Level: SPL A-weighting, SPL C-weighting, SPL (unweighted)–250 g explosive charge on the surface-Station 3.

**Figure 22:**
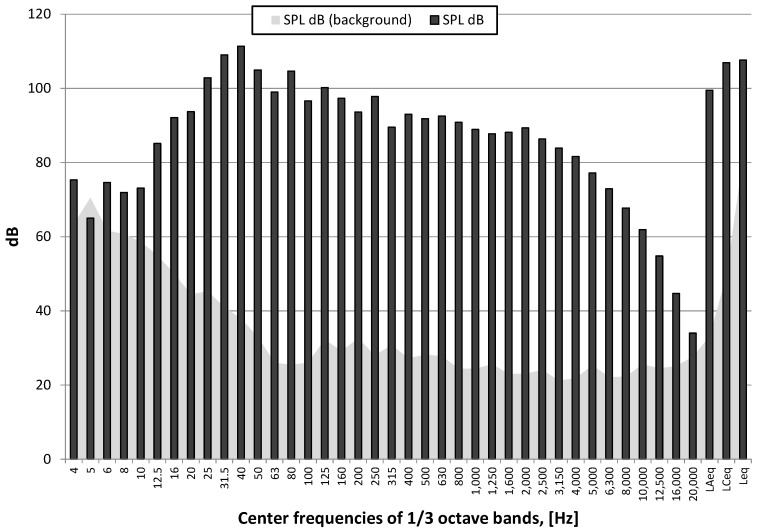
Spectrum of maximum SPL (unweighted) in one-third octave bands—250 g explosive charge on the surface and background noise (SPL dB (background))-Station 3.

Another phase involved the detonation of a series of explosive charges which were placed by different procedures (the mass of the explosive charge and its location were different—the charge was placed on the surface or buried). As before, the measurements were taken both inside and outside the building. Examples of test results are shown in Figure 20, Figure 21 and Figure 22 (the mass of the detonated explosive charge being 250 g with the charge placed on the surface; the distance to the measuring station being 90 m) and Figure 20 (the mass of detonated explosive charge being 1000 g with the charge placed in a hole; the distance to the measuring station being 90 m).

Figure 21 shows the course of the A-weighting, C-weighting, and unweighted sound pressure level [34], while Figure 22 shows the spectrum at the maximum value of the sound pressure from Figure 21.

The detonation of the explosive charge on the surface had generated an airblast wave pressure which, as in the case of the pyrotechnic charge, had an effect on the vibration meters located both inside and outside the building. The triggering time was the same in all cases. As before, the building wall dampened the airblast wave pressure. In this case, there was significant differences between the recordings inside and outside the building made by the vibration meters. Inside the building the vibration velocity is considerably lower (approx. 3 times lower). The vibration structure is dominated by high frequencies, reaching 300 Hz.

**Figure 23:**
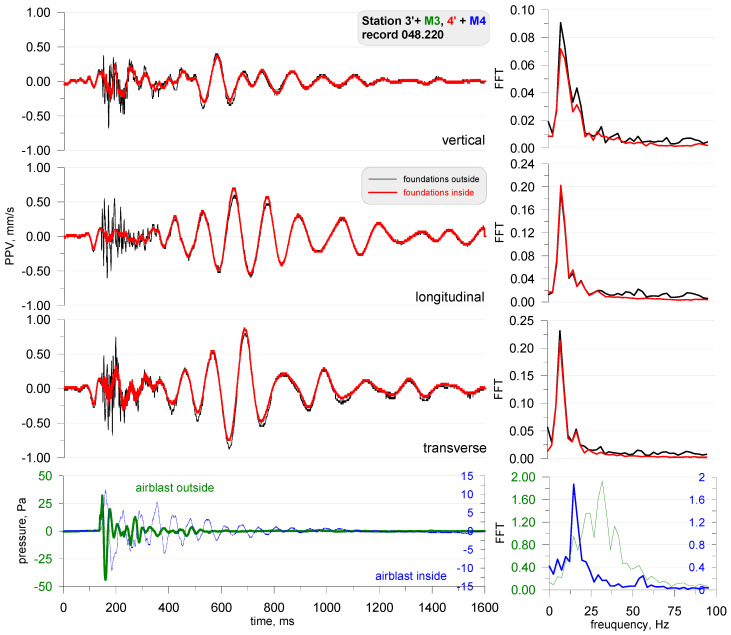
Foundation vibration seismogram (three directional components) and recordings of airblast wave pressure combined with the analysis of the FFT-Station 3 and 4—buried explosive charge.

**Figure 24:**
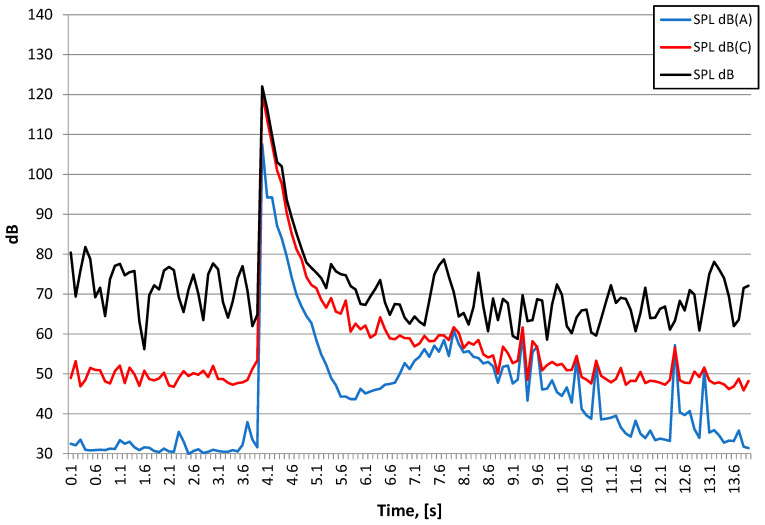
Waveforms of Sound Pressure Level: SPL A-weighting, SPL C-weighting, SPL (unweighted)—1000 g buried explosive charge-Station 3.

**Figure 25:**
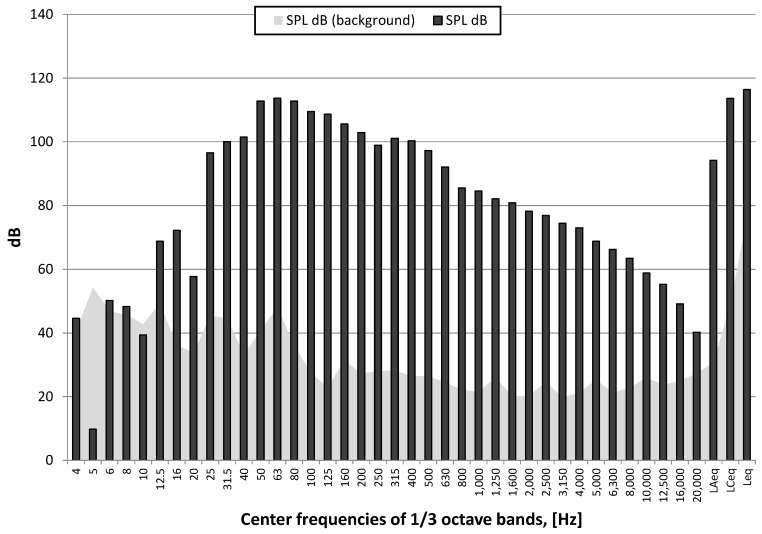
Spectrum of maximum SPL (unweighted) in one-third octave bands—1000 g buried explosive charge and background noise (SPL dB (background)-Station 3.

The findings are different for the detonation of a larger explosive charge that is placed in a hole and buried (Figure 23, Figure 24 and Figure 25). Once more, the airblast wave pressure started the recording but further on, ground vibrations were recorded by the vibration meters. Both sensors recorded the same signals, with the recordings practically overlapping. Low frequencies (approx. 10–12 Hz), typical of buildings, are prevalent in the frequency structure. It can be seen when vibrations reach the building at the beginning of the signal (t = 0.1 s), and then the airblast wave appears and interferes with the signal (t = 0.15 s–0.3 s). Another signal phase is related to surface waves that are characterised by lower propagation speed in the rock medium.

Figure 24 shows the course of the A-weighting, C-weighting, and unweighted sound pressure level [34], while Figure 25 shows the spectrum at the maximum value of the sound pressure level from Figure 24.

The nature of the acoustic wave producing an auditory event is low-frequency. For both detonation types, the energy is focused in the range of 25–100 Hz, with a 40 Hz harmonic for the 250 g charge detonated on the surface and 63 Hz for the 1000 g charge that was detonated when buried. The instantaneous peak sound levels (LCpeak) in both cases is close to 140 dB, which is approx. 200 Pa. The A-weighting sound pressure level (SPL dB(A)), measured using the time constant FAST (125 ms) for the 1000 g explosive charge buried is almost 120 dB, and is approx. 10 dB larger compared to the detonation of the 250 g explosive charge on the surface. The difference in linear scale means that the energy reaching the measuring station was more than three times higher. Thus, the burial of the explosive charge alone does not change sound energy because the mass of the explosive charge was increased four times.

## 4. Discussion

The presented examples show the possibility of recording vibration interference and what problems one may come across during the acquisition of data on signals recorded during the detonation of explosive charges that are placed and detonated in various ways. Badly performed blasting works in opencast mines may cause a significant pressure of the blast wave. It can be seen that geophones are prone to effects propagated in the air. The question then arises: does the interference occurring through air propagation affect the sensor alone or does it also affect the ground or the building recorded by geophones? The measurements carried out in the field and in the laboratory confirm the fact that the air overpressure affects the geophones. This fact can lead to an incorrect assessment of ground vibrations recorded by geophones. Its effect depends on the pressure value. The first effects of impact on geophones can be seen at a pressure of about 50 Pa.

A review of standards in several countries recommends that the sensors to collect ground vibrations need to be buried some depth into the ground or protected from the environment to avoid the collection of strange data. In this paper’s case, the vibration sensors were exposed to the air, recording the pass of the air overpressure (there are no guidelines in the Polish standard as to how to install the sensor in the ground; therefore, it was mounted following the requirements of the apparatus manufacturer using original accessories).

In the case of vibration measurements performed in construction objects, the impact of the air overpressure on the sensor itself (mounted outside—Figure 11, Figure 19, Figure 20 and Figure 23) and on the building itself (this is confirmed by the measurements shown in Figure 7 and Figure 20, where the sensor was mounted inside a closed building). The measurements show that the air overpressure in the range of 120–150 Pa causes vibrations of the building. This information is relevant from the point of view of assessing the effect of vibrations on buildings as this assessment is based on the idea that vibrations are propagated through the ground and not through the air. Therefore, it may turn out that such an assessment may result in the misinterpretation of results.

The above observations confirm the necessity to measure the air overpressure while measuring the impact of vibrations generated by various types of works in which the explosive is used. This is especially true when the explosive is detonated on the surface, or the stemming is not large, or shaped charges are used.

Interestingly, in the last field example (Figure 17) it was demonstrated that airblast wave microphones recording sound in rooms were generated as a result of material vibrations in the building and the transmission of sound energy. One of the hypotheses that is to be considered is that the microphone diaphragm started to vibrate due to the vibrations transmitted to the microphone stand through the ground. This is observed for small frequencies (below 31.5 Hz) when microphones are calibrated, i.e., when frequency responses describing sensitivity are determined using electrostatic actuators according to IEC 61094-6:2004.

This paper constitutes the first part of the results of the study aimed to show how difficult it is to acquire and interpret measurement data recorded during the detonation of explosive materials. The results of the tests carried out in a reverberation chamber as well as the results of filtering analysis that eliminates air-propagated interference will be presented in the next paper [35], so it is possible to install the sensor without a shield, but it requires simultaneous measurement of vibration and pressure of the air shock wave.

## 5. Conclusions

Based on the conducted research and preliminary analysis, it can be concluded that the problem of measuring the impact of vibrations and air shock waves resulting from the detonation of explosive charges is a difficult issue and requires extensive experience, both when performing tests and analysing their results. Due to the more and more frequent execution of blasting works in the vicinity of buildings, it is necessary to apply new methods of vibration forecasting [36] or their analysis [12,35,37]. Thanks to this, it is possible to increase the credibility of the forecast and at the same time to a greater degree control the negative impact to environment of the blasting works performed.

## Figures and Tables

**Table 1:** Technical specifications of UVS 1608.

Parameter	Range
Frequency range	2–250 Hz (−3 dB)
Sampling frequency	User selectable: 100, 500, 1000, 2000, or 4000 Hz
Measuring range	±250 mm/s
Resolution	Better than: 0.02 mm/s up to 31 mm/s, Better than 0.1 mm/s up to 250 mm/s
Threshold increment	0.01 mm/s

**Table 2:** Technical specification of microphones.

Parameter	Range
Sensitivity	2 mV/Pa
Frequency range	2–8000 Hz (−3 dB)
Dynamic range	Input: 1500 Pa (158 dB linear relative 20 μPa) Output: ±3 V

**Table 3:** Technical specification of geophones.

Parameter	Range
Sensitivity	20 mV/mm/s
Frequency range	1–1000 Hz (−3 dB at 1 Hz)
Resonant frequency	4.5 Hz, ±0.5 Hz (undamped)
Dynamic range	±2 mm displacement corresponding to: (a) 50 mm/s within 1–4 Hz (b) 100 mm/s within 8–1000 (c) linear in between
